# Morning Heart Rate Variability as an Indication of Fatigue Status in Badminton Players during a Training Camp

**DOI:** 10.3390/sports8110147

**Published:** 2020-11-10

**Authors:** Taro Iizuka, Nao Ohiwa, Tomoaki Atomi, Miho Shimizu, Yoriko Atomi

**Affiliations:** 1 Material Health Science Laboratory, Graduate School of Engineering, Tokyo University of Agriculture and Technology, Tokyo 184-8588, Japan; t-iizuka@badminton.or.jp (T.I.); mshmz@cc.tuat.ac.jp (M.S.); 2 Nippon Badminton Association, Tokyo 160-0013, Japan; 3 Department of Sports Research, Japan Institute of Sports Sciences, Tokyo 115-0056, Japan; nao.ohiwa@jpnsport.go.jp; 4 Department of Physical Therapy, Faculty of Health Sciences, Kyorin University, Tokyo 181-8612, Japan; tatomi@ks.kyorin-u.ac.jp

**Keywords:** heart rate variability, badminton, fatigue, physical condition, autonomic nervous activity

## Abstract

This study aimed to clarify whether changes in the fatigue status of elite athletes during a precompetition period could be evaluated using morning heart rate variability (HRV) indices. Eight Japanese National Badminton Team players (age, 23.0 ± 2.8 years) participated in this study. HRV and subjective fatigue were measured during the first (days 1–4: Phase 1) and the second half (days 5–8: Phase 2) of an 8-day national team training camp. The global and parasympathetic HRV indices were as follows: standard deviation of all R-R intervals (SDNN) (Phase 1, 87.5 ms; Phase 2, 104.3 ms; *p* < 0.05), root mean square of the successive R-R interval differences (RMSSD) (Phase 1, 66.6 ms; Phase 2, 103.6 ms; *p* < 0.05), and high-frequency component power (HF) (Phase 1, 1412.0 ms^2^; Phase 2, 3318.5 ms^2^; *p* < 0.05). All the aforementioned indices increased significantly from Phase 1 to Phase 2. Significant correlations were observed between the change in subjective fatigue and changes in SDNN, RMSSD, and HF (ρ = −0.80, *p* = 0.017; ρ = −0.77, *p* = 0.027; and ρ = −0.80, *p* = 0.017, respectively). Measuring morning HRV indices may be effective for objectively evaluating changes in the fatigue status of elite athletes during a precompetition period.

## 1. Introduction

Excessive training during a precompetition period can induce fatigue accumulation, which may lead to impaired performance in subsequent competitions. Therefore, during the precompetition period, training load should be adequately controlled to ensure that fatigue is reduced when athletes participate in a competition [1]. However, establishing a tool to objectively monitor how athletes respond to training would be necessary to guide training to optimize their performance, as their response to training may vary [2,3].

Monitoring heart rate variability (HRV) is a noninvasive method that enables the evaluation of cardiac autonomic nervous activity [4,5]. Previous studies have shown that monitoring the HRV to evaluate the cardiac parasympathetic nervous activity alone or in combination with the sympathetic nervous activity is effective for objectively monitoring the athletes’ fatigue status [5,6,7,8]. Therefore, monitoring individual HRV may provide an objective measure to evaluate changes in the athlete’s fatigue status during the precompetition period.

Our purpose was to clarify whether changes in the fatigue status of elite athletes during a precompetition period could be objectively evaluated by measuring individual HRV indices. We examined the relationships between the HRV indices and subjective fatigue in elite badminton players during a training camp in preparation for an international tournament. We hypothesized that the changes in the HRV indices would significantly correlate with changes in players’ subjective fatigue status during a training camp.

## 2. Materials and Methods

### 2.1. Participants

Eight internationally ranked badminton players (age, 23.0 ± 2.8 years; sex, four women and four men; world ranking position, 41 ± 16), who were members of the Japan National Team, participated in the study. Athletes who completed the entire training program during a national team training camp and subsequently competed the following week in the Japan Open 2013 were included in this study. All participants were fully informed regarding the purpose and procedures of this study, and their informed consent to participate in the study was obtained. This study was approved by the Human Subjects Committee of the Japan Institute of Sports Sciences (approval no. 023 in 2013) and conducted in accordance with the tenets of the Declaration of Helsinki.

### 2.2. Procedures

The study was conducted during an 8-day national team training camp held at the national training center (Tokyo, Japan) in preparation for the badminton Japan Open 2013 that was held in Tokyo immediately after the training camp. The Japan Open 2013 was one of the 12 Badminton World Federation Super Series tournaments, which were widely regarded as badminton competitions with the highest standards worldwide [9]. Compared with the first half of the training camp (days 1–4: Phase 1), the training load during the second half of the training camp (days 5–8: Phase 2) was planned to reduce players’ fatigue for the Japan Open, mainly by decreasing the training volume while maintaining training intensity. HRV and subjective fatigue data were collected every morning at a similar time of the day (between 6:00 a.m. and 8:00 a.m.) to avoid the influence of circadian variations on HRV indices.

### 2.3. Measurements

#### 2.3.1. Training Duration

The daily training program consisted of morning and afternoon sessions, each lasting for 2 or 3 h. In addition to training at the badminton hall, a 2-h resistance training at their training gym was included in the afternoon training session on days 1, 3, and 5. On days 4 and 8, no training session was held in the afternoon. As a measure of the daily training volume, the daily training duration was calculated by tallying the durations of the morning and afternoon sessions daily.

#### 2.3.2. Training Intensity

During the training at the badminton hall, heart rate (HR) was recorded using a Polar RS800CX heart rate monitor (Polar Electro Oy, Kempele, Finland). As a measure of daily training intensity, the mean HR and maximum HR during training were evaluated daily for each player.

#### 2.3.3. HRV

Following a rest period of at least 1 min, the beat-to-beat HR was recorded in a seated position for 5 min using a Polar RS800CX heart rate monitor (Polar Electro Oy, Kempele, Finland) under a controlled breathing rate of 15 breaths per min. Data were processed using Kubios HRV analysis software (Kubios HRV Standard, ver. 3.0.2., Kubios, Kuopio, Finland) with time and frequency domain analyses [4]. Concerning the time domain HRV indices, we analyzed the standard deviation of all R-R intervals (SDNN) and the square root of the mean squared differences of successive R-R intervals (RMSSD). SDNN reflects parasympathetic and sympathetic activities, while RMSSD reflects parasympathetic cardiac modulation [4,5]. Concerning the frequency domain HRV indices, we analyzed the absolute power of low-frequency (LF: 0.04–0.15 Hz) and high-frequency (HF: 0.15–0.40 Hz) band components. Additionally, the normalized HF unit (HFnu; HF/(LF + HF) × 100) and the LF/HF ratio were analyzed. LF represents the parasympathetic and sympathetic activities, and HF represents the parasympathetic activity [10]. HFnu and the LF/HF ratio represent evaluations of the autonomic nervous system balance [4,10].

#### 2.3.4. Subjective Fatigue

Subjective fatigue was estimated using a 100-mm visual analogue scale. The participants were asked to indicate their degree of fatigue by marking the 100-mm line between 0 (no fatigue) and 100 (maximum fatigue) [11].

### 2.4. Statistical Analysis

For mean HR, maximum HR, HRV indices, and subjective fatigue, data were collected for 4 days each in Phases 1 and 2, and their means were used for further analyses. A normality test was performed using the Shapiro–Wilk test. Considering the skewed distribution of the data and the small number of participants, the Wilcoxon signed-rank test was used to determine significant differences between the phases in each variable. The data are presented as medians and interquartile ranges. The effect sizes were determined using the coefficient r [12]. The interpretation of the coefficient r is as follows: r = 0.1: small, 0.3: moderate, 0.5: large, 0.7: very large, and 0.9: extremely large [13]. Spearman’s rank rho (ρ) correlation coefficient was calculated to examine the relationships between the change in subjective fatigue and the changes in the HRV indices from Phase 1 to Phase 2. Statistical significance was set at *p* < 0.05. All statistical analyses were performed using SPSS version 19 (IBM Corp., Armonk, NY, USA).

## 3. Results

### 3.1. Training Duration

Daily training duration decreased from 5.3 ± 1.5 h in Phase 1 to 3.8 ± 1.3 h in Phase 2.

### 3.2. Training Intensity

A significant decrease in the mean HR was observed between Phase 1 and Phase 2 (Phase 1: 130.4 beats/min (128.1–134.9); Phase 2: 111.5 beats/min (108.9–116.6); *p* = 0.012; r = 0.89). Furthermore, a significant decrease was observed in the maximum HR between Phase 1 and Phase 2 (Phase 1: 179.9 beats/min (177.7–189.2); Phase 2: 164.9 beats/min (162.4–173.4); *p* = 0.012; r = 0.89).

### 3.3. HRV

Table 1 shows the differences in HRV indices between Phase 1 and Phase 2. The HR decreased significantly from Phase 1 to Phase 2 (*p* = 0.012). Regarding time domain HRV indices, SDNN and RMSSD increased significantly from Phase 1 to Phase 2 (*p* = 0.012 and *p* = 0.012, respectively). Regarding frequency domain HRV indices, LF and HF increased significantly from Phase 1 to Phase 2 (*p* = 0.012 and *p* = 0.012, respectively), while no significant difference was observed in HFnu and LF/HF (*p* = 0.401 and *p* = 0.575, respectively).

### 3.4. Subjective Fatigue

**Figure 1:**
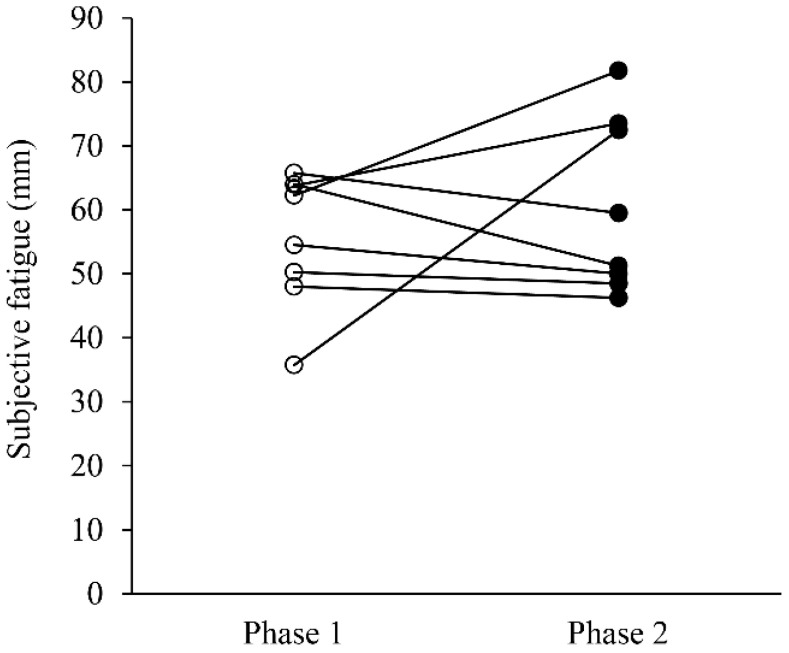
Comparison of subjective fatigue between Phase 1 and Phase 2.

Figure 1 shows the differences in subjective fatigue between Phase 1 and Phase 2. No significant difference was observed in subjective fatigue between Phase 1 and Phase 2 (Phase 1: 58.4 mm (48.6–63.9); Phase 2: 55.4 mm (48.9–73.3); *p* = 0.779; r = 0.10).

### 3.5. Relationships between Changes in Subjective Fatigue and Changes in HRV

**Figure 2:**
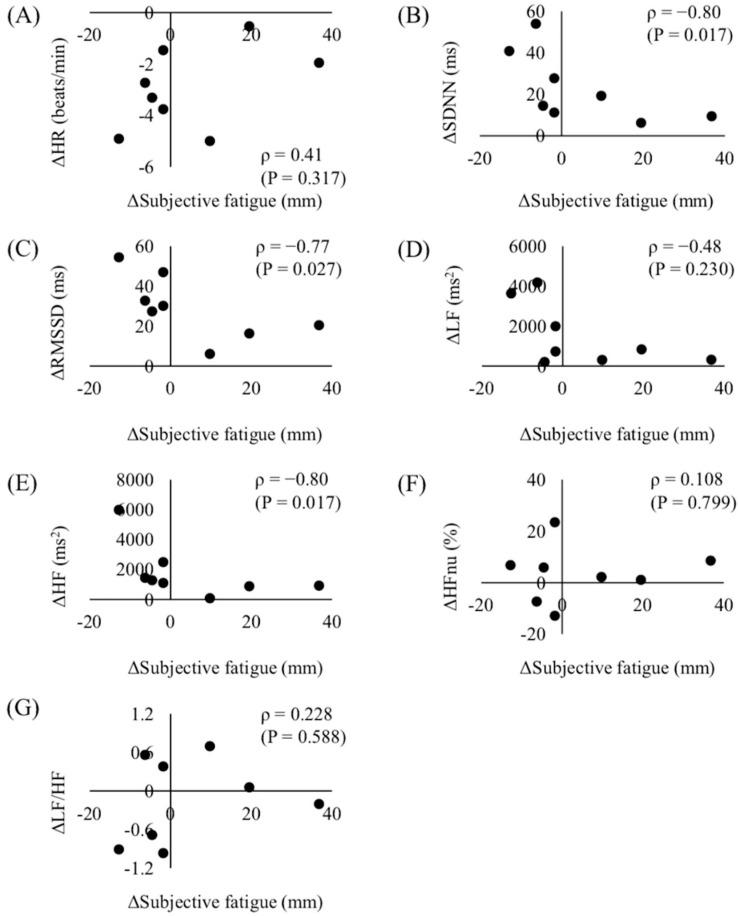
Relationships between the changes in subjective fatigue and the changes in heart rate variability (HRV) indices. (**A**) HR, (**B**) SDNN, (**C**) RMSSD, (**D**) LF, (**E**) HF, (**F**) HFnu, and (**G**) LF/HF from Phase 1 to Phase 2. HRV: heart rate variability; HR: heart rate; SDNN: standard deviation of all R-R intervals; RMSSD: square root of the mean squared differences of successive R-R intervals; LF: low-frequency component power of HRV; HF: high-frequency component power of HRV; HFnu: normalized unit of HF; LF/HF: ratio of LF and HF.

Figure 2 shows the relationships between the changes in subjective fatigue and HRV indices from Phase 1 to Phase 2.

## 4. Discussion

The purpose of this study was to clarify whether the change in the fatigue status of elite athletes during a precompetition period could be objectively evaluated by measuring the HRV indices. Considering the decrease in the mean HR and maximum HR from Phase 1 to Phase 2 due to the decrease in training duration and intensity, significant changes were observed in the HRV indices. We demonstrated that the changes in HRV indices, such as SDNN, RMSSD, and HF, significantly correlated with the change in subjective fatigue. To the best of our knowledge, this is the first study to practically demonstrate that changes in the fatigue status of elite badminton players could be evaluated by measuring morning HRV indices.

Changes in training load have been shown to alter the HRV indices [10,14,15,16,17,18,19,20,21,22]. Pichot et al. [10] examined the relationship between HRV and training load in middle-distance runners and indicated that SDNN and RMSSD increased significantly with the decrease in training load during the recovery week. The authors suggested that the increases in SDNN and RMSSD reflect an increase in global HRV, associated with a relative increase in the parasympathetic drive. Besides, Iellamo et al. [15] showed that a significant decrease in HF was induced by an increase in training load in elite junior rowers. Considering the results from previous studies, the increases in SDNN, RMSSD, and HF during the training camp observed in our study may be induced by the decreased training duration and intensity from Phase 1 to Phase 2 to prepare the athletes for the Japan Open held immediately after the training camp.

However, although previous studies have shown that an increase in parasympathetic HRV indices is related to decreased fatigue status in athletes [10,14,23,24,25], subjective fatigue did not decrease significantly despite the increase in parasympathetic HRV indices. Several studies have examined the HRV response of elite athletes during a training camp immediately before important competitions to assess the possibility of fatigue accumulation [26,27]. Nakamura et al. [26] reported that elite karate athletes did not present signs of fatigue accumulation, as relatively steady HRV was recorded during the training camp. In contrast to the findings by Nakamura et al. [26], where moderate training loads were imposed on the athletes throughout the training camp, relatively high training volumes and intensities were imposed on the players in the first-half of the training camp (Phase 1) in our study. Our results indicate that the training load decrease from Phase 1 to Phase 2 was not sufficient to reduce the players’ fatigue status. A reduction in the fatigue status of the players may have been a challenge despite the decreased training load in Phase 2 because of the high impact of the training load in Phase 1. An increase in subjective fatigue status from Phase 1 to Phase 2 was observed in three participants.

In practice, it is important to evaluate individual responses to an altered training load. Flatt et al. [28] examined the relationship between the training load and HRV in female collegiate soccer players and suggested that parasympathetic HRV responses to varying training loads were highly individual because of the differences in individual fitness and fatigue status. We demonstrated that changes in SDNN, RMSSD, and HF significantly correlated with the change in subjective fatigue. Therefore, our results suggest that measuring the global and parasympathetic HRV indices, such as SDNN, RMSSD, and HF, may be effective for objectively evaluating changes in the fatigue status of badminton players on an individual basis. Recently, smartphone applications that allow easy and brief evaluation of parasympathetic HRV responses are utilized among athletes and coaches [29,30]. The use of these applications may practically guide the effective control of the training load during a training camp to optimize the performance of each player in subsequent tournaments. Further research is warranted to examine whether measuring the global and parasympathetic HRV indices is effective for objectively evaluating changes in the fatigue status of badminton players in different training phases.

This study has several limitations. First, since we aimed to investigate changes in the fatigue status of international-class badminton players who completed the entire training program during the training camp, we could only recruit eight participants for this study. In this study, six out of eight participants were doubles players. As the physical demands of singles and doubles disciplines in badminton differ from each other [31,32,33,34], the extent to which HRV indices reflect changes in the players’ fatigue status may be different between singles and doubles players. Second, we could not evaluate the players’ overall daily training load since we could not monitor the training intensity during their resistance training sessions by measuring HR. Quantifying the overall training load including the resistance training sessions may further enhance our understanding of how the fatigue status of each player is altered by training during a training camp.

In conclusion, we demonstrated that changes in HRV indices significantly correlated with the change in the subjective fatigue of international-class badminton players during a training camp. These results suggest that measuring the HRV indices may be effective for objectively evaluating changes in the fatigue status of international-class badminton players on an individual basis. Therefore, information obtained from HRV monitoring could be used to prevent the accumulation of fatigue, leading to the optimization of the performance of badminton players. Measuring HRV indices may be utilized more generally for evaluating the fatigue status that may vary between individuals.

## Figures and Tables

**Table 1:** Comparisons of heart rate variability indices between Phase 1 and Phase 2.

	Phase 1	Phase 2	*p*-Value	Effect Size
HR (beats/min)	55.6 (52.5–63.4)	53.5 (50.8–59.2)	0.012	0.89
SDNN (ms)	87.5 (71.6–103.8)	104.3 (95.3–120.3)	0.012	0.89
RMSSD (ms)	66.6 (51.2–82.7)	103.6 (81.1–114.5)	0.012	0.89
LF (ms^2^)	1933 (758–3069)	2949 (2448–4146)	0.012	0.89
HF (ms^2^)	1412 (1081–2246)	3318 (2218–4439)	0.012	0.89
HFnu (%)	42.1 (38.6–54.2)	49.4 (35.9–62.6)	0.401	0.30
LF/HF	1.52 (1.48–1.78)	1.19 (0.68–1.98)	0.575	0.20

Data are presented as medians and interquartile ranges. HR: heart rate; SDNN: standard deviation of all R-R intervals; RMSSD: square root of the mean squared differences of successive R-R intervals; LF: low-frequency component power of heart rate variability; HF: high-frequency component power of heart rate variability; HFnu: normalized unit of HF; LF/HF: ratio of LF to HF.
